# Controlling Doxorubicin Release from a Peptide Hydrogel through Fine-Tuning of Drug–Peptide Fiber Interactions

**DOI:** 10.1021/acs.biomac.2c00356

**Published:** 2022-05-11

**Authors:** Mohamed A. Elsawy, Jacek K. Wychowaniec, Luis A. Castillo Díaz, Andrew M. Smith, Aline F. Miller, Alberto Saiani

**Affiliations:** † Department of Materials, University of Manchester, Oxford Road, Manchester M13 9PL, U.K.; ‡ Manchester Institute of Biotechnology, Oxford Road, Manchester M13 9PL, U.K.; § Department of Chemical Engineering and Analytical Sciences, University of Manchester, Oxford Road, Manchester M13 9PL, U.K.

## Abstract

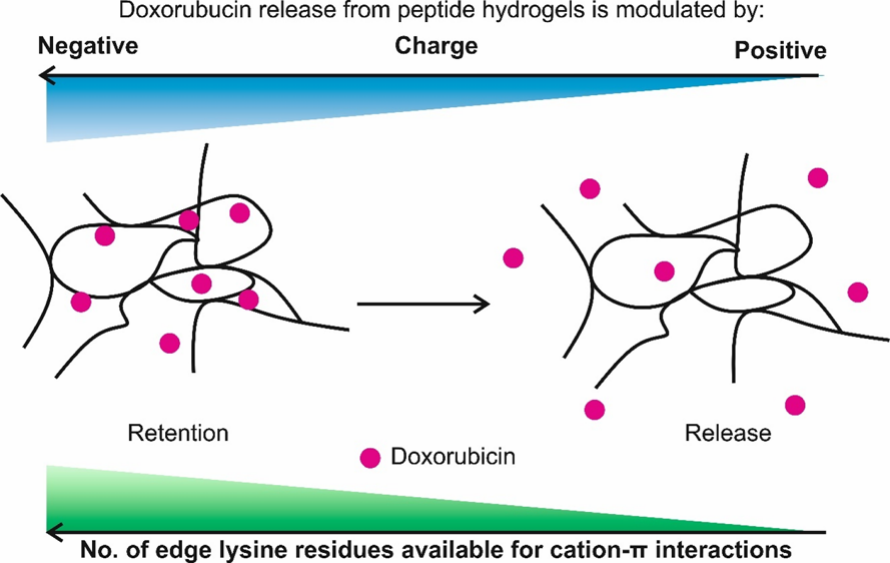

Hydrogels are versatile materials that have emerged in the last few decades as promising candidates for a range of applications in the biomedical field, from tissue engineering and regenerative medicine to controlled drug delivery. In the drug delivery field, in particular, they have been the subject of significant interest for the spatially and temporally controlled delivery of anticancer drugs and therapeutics. Self-assembling peptide-based hydrogels, in particular, have recently come to the fore as potential candidate vehicles for the delivery of a range of drugs. In order to explore how drug–peptide interactions influence doxorubicin (Dox) release, five β-sheet-forming self-assembling peptides with different physicochemical properties were used for the purpose of this study, namely: FEFKFEFK (F8), FKFEFKFK (FK), FEFEFKFE (FE), FEFKFEFKK (F8K), and KFEFKFEFKK (KF8K) (F: phenylalanine; E: glutamic acid; K: lysine). First, Dox-loaded hydrogels were characterized to ensure that the incorporation of the drug did not significantly affect the hydrogel properties. Subsequently, Dox diffusion out of the hydrogels was investigated using UV absorbance. The amount of drug retained in F8/FE composite hydrogels was found to be directly proportional to the amount of charge carried by the peptide fibers. When cation−π interactions were used, the position and number of end-lysine were found to play a key role in the retention of Dox. In this case, the amount of Dox retained in F8/KF8K composite hydrogels was linked to the amount of end-lysine introduced, and an end-lysine/Dox interaction stoichiometry of 3/1 was obtained. For pure FE and KF8K hydrogels, the maximum amount of Dox retained was also found to be related to the overall concentration of the hydrogels and, therefore, to the overall fiber surface area available for interaction with the drug. For 14 mM hydrogel, ∼170–200 μM Dox could be retained after 24 h. This set of peptides also showed a broad range of susceptibilities to enzymatic degradation opening the prospect of being able to control also the rate of degradation of these hydrogels. Finally, the Dox released from the hydrogel was shown to be active and affect 3T3 mouse fibroblasts viability in vitro. Our study clearly shows the potential of this peptide design as a platform for the formulation of injectable or sprayable hydrogels for controlled drug delivery.

## Introduction

Hydrogels are versatile materials that have emerged in the last few decades as promising candidates for a range of applications in the biomedical field, from tissue engineering and regenerative medicine to controlled drug delivery.^1^ In the drug delivery field, in particular, they have been the subject of significant interest for the spatially and temporally controlled delivery of anticancer drugs and therapeutics. Indeed, delivery of chemotherapy through systemic routes is associated with significant side effects, and hydrogel-based local delivery vehicles are thought to have the potential to provide significant benefits by increasing drug efficacy through local targeting of tumors and reduce off-target toxicity.^2^

**Figure 1:**
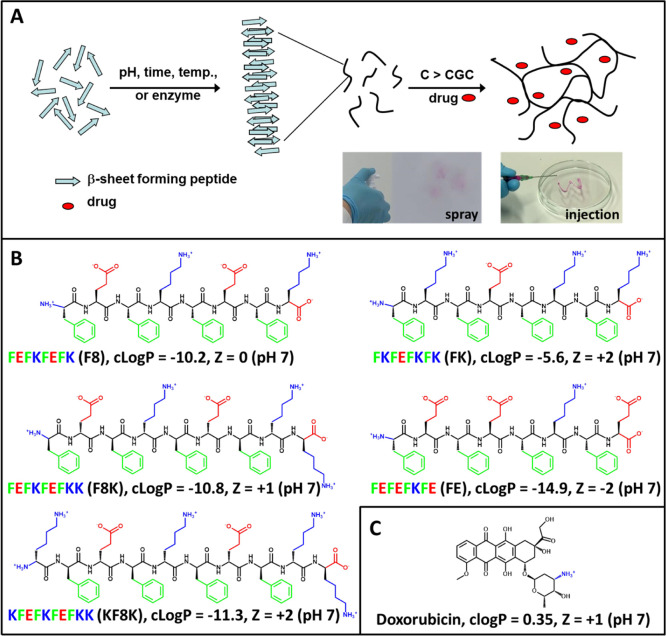
(A) Schematic representation of the self-assembly and gelation pathways of β-sheet-forming peptides. Photographs illustrating spraying and injecting of peptide hydrogels; (B) Chemical structures of peptides used in this study and selected physicochemical properties at pH 7; (C) Chemical structure and selected physicochemical properties of Dox at pH 7.

In this context, self-assembling peptide-based hydrogels have recently come to the fore as potential candidate vehicles for the delivery of a range of drugs.^3^ They have indeed been used as carriers for both therapeutic proteins^4^ and small drug molecules.^5^ Their unique properties make them ideal as injectable materials for an in vivo localized drug delivery. They can be designed to have an excellent biocompatibility, a low immunogenicity, and unique shear thinning and recovery properties, eliminating the need for postinjection crosslinking triggering and/or chemistry. In addition, these unique mechanical properties make the incorporation of the drug cargo in these materials, and therefore their formulation, straightforward through simple mixing. A number of self-assembling peptide designs that form stable hydrogels can be found in the literature.^6^ In our group, we have developed, in the past two decades, a platform for the design of peptide hydrogels based on a family of amphipathic short peptides (typically 8 to 12 amino acids long) with alternating hydrophilic and hydrophobic residues based on the early study of Zhang and co-workers.^7^ This family of peptides has been shown to readily self-assemble into antiparallel β-sheet rich fibers that entangle/associate into dense 3D fibrillar networks, forming very stable injectable and sprayable hydrogels (Figure 1A).^4d,5a,5e,8,9^

One of the key challenges when controlling the delivery of small oncologic drugs are the interactions with the hydrogel fibrillar network. Indeed, diffusion of small drugs (size significantly smaller than mesh size of the network) out of hydrogels is directly linked to the type and strength of these interactions.^2a,4a,5b,5e,8d^ Their exact nature will depend on the drug and self-assembling peptide chemistries.^10^ Oncologic drugs are typically chemically complex molecules with the ability to have diverse molecular interactions. A significant fraction of drug compounds is either aromatic or contains aromatic moieties that are, in most cases, key to their pharmacological activity. These aromatic moieties bind to the biological macromolecular receptor targeted (e.g., proteins, enzymes, and nucleic acids) through various intermolecular interactions, including cation−π and π–π.^11^ Both these interactions are also known to be involved in protein structural stabilisation,^12^ protein–protein interfaces,^13^ and biological recognition.^14^ Self-assembling peptide design, on the other hand, can be tailored through the incorporation of selected amino acids to introduce specific interaction capabilities, such as hydrogen bonding, hydrophilic/hydrophobic, electrostatic, cation−π, and π–π, by exploiting the 20 natural amino acid library functional diversity. In addition, non-natural amino acids can also be designed to introduce specific functionalities and, therefore, interaction capabilities. As a result, self-assembling peptide hydrogels offer a flexible platform for the design of drug delivery systems.

Recently, we showed how electrostatic interactions between peptide fibers and two small, charged model compounds could be used to control their diffusion.^5e,8d^ In the present study, we decided to extend our investigation to understand how cation−π and π–π interactions can be used to control the delivery of a common oncologic drug, doxorubicin (Dox). Dox is a widely clinically used aromatic chemotherapeutic agent with a broad spectrum of activity against various tumor types. It is also widely used as a theragnostic agent as it is fluorescent and UV absorbent. As most oncologic drug, Dox has significant side effects when delivered systemically, in particular cardiotoxicity, that limit its clinical use.^15^ Controlled localized delivery of Dox is seen as a potential approach to decrease these side effects and extend its use.^16^

In order to explore how drug-peptide interactions influence Dox release, five β-sheet-forming self-assembling peptides with different physicochemical properties were used for the purpose of this study: namely FEFKFEFK (F8), FKFEFKFK (FK), FEFEFKFE (FE), FEFKFEFKK (F8K), and KFEFKFEFKK (KF8K) (F: phenylalanine; E: glutamic acid; K: lysine). Their chemical structures and properties are presented in Figure 1B. All of them contain lysine and phenylalanine and, therefore, have the ability to form cation−π and π–π interactions. In addition, as Dox carries a positive charge at pH 7 (Figure 1C), peptides with a range of charges were selected. Assuming that the p*K*_a_ of the ionic side and terminal groups are not significantly affected by the self-assembly, FE will carry at pH 7 a negative −2 charge while FK, F8K, and KF8K will carry positive charges of +2, +1, and +2, respectively, and F8 will carry an overall neutral charge. First, Dox-loaded hydrogels were characterized to ensure that the incorporation of the drug did not significantly affect the hydrogel properties. Subsequently, Dox diffusion out of the hydrogels was investigated using UV absorbance.

## Materials and Methods

### Materials

Peptides were purchased as HCl salts from Biomatik Corporation (Wilmington, DE, Canada). The peptide purities (>95%) were confirmed in-house by mass spectroscopy (MS) and reverse-phase high-performance liquid chromatography (HPLC). All solvents and reagents were purchased from Sigma-Aldrich and used as received.

### Hydrogel Formulation

Unloaded and loaded hydrogels were prepared by suspending the required amount of peptide powder in 350 μl of doubly distilled water (ddH_2_O) or 200 μg mL^–1^ Dox ddH_2_O solution, respectively. The suspensions were sonicated (80 kHz) and vortexed until full dissolution was achieved. The solution pH was then adjusted to 5.0–5.7 by stepwise addition of 0.5 M NaOH solution to trigger gelation. Finally, the hydrogel volumes were adjusted to 500 μL by the addition of ddH_2_O. The samples were vortexed after each NaOH and ddH_2_O addition to ensure homogenous mixing. If bubbles were present, the samples were gently centrifuged to remove all trapped air bubbles. All hydrogels were formulated at a final peptide concentration of 14 mM, and drug-loaded hydrogels were formulated at a final Dox concentration of 240 μM. Formulations were stored at 4 °C overnight before use.

### Attenuated Total Reflectance–Fourier Transform Infrared Spectroscopy

Hydrogels were spread as prepared onto the crystal surface of a Bruker ALPHA-P Fourier transform infrared spectroscopy (FTIR) spectrometer equipped with a diamond multibounce attenuated total reflectance (ATR) plate. The transmittance spectra were recorded (128 scans) between 4000 and 400 cm^–1^ with a resolution of 4 cm^–1^. HPLC grade water was used as background and was automatically subtracted from the recorded spectra using OPUS software provided with the instrument.

### Oscillatory Rheology

Rheological studies were carried out on a stress-controlled rheometer (Discovery HR-2, TA Instruments) equipped with a solvent trap to minimize evaporation using a 20 mm parallel plate geometry. 500 μL of the peptide hydrogel was loaded onto the stage, and the gap between the stage and the upper plate was set to 250 μm. The loaded sample was then left for 2 min to equilibrate at 37 °C before measurement. The excess sample was carefully removed with a spatula from around the plate. Frequency sweeps were measured between 0.01 and 15 Hz, and a strain of 0.1%. All measurements were repeated at least three times.

### Drug Release

Dox release from hydrogels was measured by UV absorbance at 485 nm and 37 °C. 1 mL release buffer, either phosphate buffer solution (PBS) or 50% (v/v) fetal bovine serum (FBS) ddH_2_O solution, was added on the top of 500 mL of hydrogels (an 8 mm thick hydrogel layer). At selected time points (2, 5, 10, 24, 48, and 72 h), the release buffer on the top of the hydrogel was very gently stirred with the tip of the pipette to ensure homogeneity, and then half of it was collected. The Dox concentration was measured using a Dox UV absorbance standard calibration curve corrected against a blank buffer. The collected release buffer was then returned to the top of the hydrogel.

### Raman Spectroscopy

Raman spectra were measured using a Renishaw inVia microscope equipped with a red laser (excitation radiation 632.8 nm) and a 600 grating, giving a spectral resolution of ∼2.6 cm^–1^. The laser power was set to 100%. 3 mL of the hydrogel or solution was gently deposited in a cell culture dish (*d* = 35 mm), resulting in a sample thickness of 6 mm. First, a wide-depth scan was performed to locate the surface of the sample. Then, depth spectra were collected going from the top of each sample in 5 μm steps in the downward direction using 10 s exposure time and a total of 5 accumulations per step. Cosmic rays were removed, and the final presented spectra were taken as the average of 10 consecutive measurements corresponding to the probing of a 50 μm sample depth. Spectra were corrected for the baseline and smoothed in Wire software (version 4.1).

### Gels Biodegradability

Peptide hydrogels (14 mM) were incubated in either PBS or 50% FBS ddH_2_O solution at 37 °C. At selected time points (2, 5, 10, 24, 48, and 72 h), the gels were disassembled by dilution to 1 mg mL^–1^ in a 1% trifluoroacetic acid (TFA) water/acetonitrile (50/50 v/v) solution. Reversed phase-high-performance liquid chromatography (RP-HPLC) analyses were conducted at 25 °C on an Ultimate 3000 HPLC system (Dionex) equipped with a variable wavelength UV detector (wavelength used 210 nm) and a gradient pump. Separation was performed on a Phenomenex Jupiter 4μ Proteo column 90A° (250 × 4.66 mm) fitted with a 300 Guard Cartridge (4.3 × 10 mm). A flow rate of 1 mL min^–1^ was used for all separations. The mobile phase consisted of a mixture of water: TFA(0.1%)—solvent A and acetonitrile/TFA(0.1%)—solvent B. An elution gradient of 90% solvent A/10% solvent B to 30% solvent A/70% solvent B over 45 min was used. 100 μL of sample aliquots was injected into the column using an ACC-3000 autosampler. Data were analyzed using Chromoleon 6.80 software. The peptide stability was expressed as a fraction of intact peptide present in the sample at sampling time *t* using the following equation
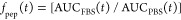
1where AUC_PBS_ (*t*) and AUC_FBS_ (*t*) are the intact peptide peak areas under the HPLC curves obtained at the sampling time *t* for hydrogels incubated with PBS and 50% FBS ddH_2_O solutions, respectively.

### Cell Culture

3T3 murine fibroblasts were seeded (1.5 × 10^4^ cells cm^–2^) in 12-well cell culture plates and 1.5 mL of DMEM supplemented with 10% FBS added per well. 400 μl of hydrogels (14 mM) with and without Dox (240 μM) were plated within 12-well cell culture plate inserts (ThinCert, Greiner Bio-One, pore size 1 μm) and placed above the cells. At selected time points, cells were retrieved from the cell culture wells, and the amount of double-stranded DNA (dsDNA) content was measured using PicoGreen dsDNA assay (Life Technologies, Carlsbad, CA, USA) following the manufacturer’s protocol.

## Results and Discussion

For the purpose of this study, all samples were formulated at 14 mM peptide concentration, at which all form stable self-supporting hydrogels. A Dox loading of 240 μM was chosen as it allowed us to investigate the nature of the interactions between the drug and the peptide fibers and highlight their effect on the release of the drug from the hydrogels over a 72 h time period.

**Figure 2:**
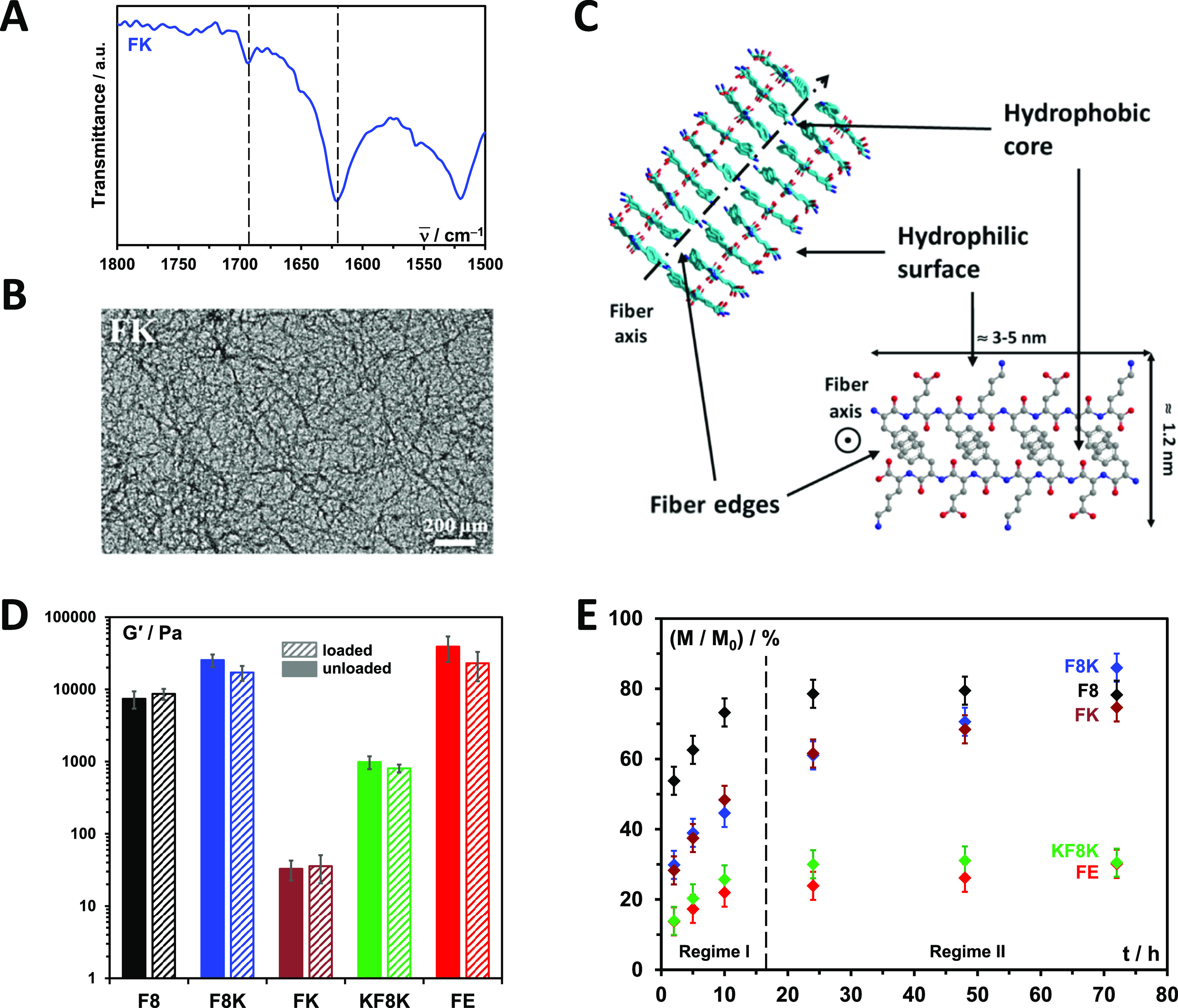
(A) FTIR spectra obtained for the FK hydrogel formulated at 14 mM peptide concentration (dotted lines indicate the position of the two bands characteristic of adoption by peptides of β-sheet conformations); (B) representative TEM image obtained for diluted FK hydrogel; (C) schematic representation of the β-sheet-rich fiber structural features formed by this family of peptides (F8 peptide shown); (D) storage shear moduli (*G*′) obtained for hydrogels formulated at 14 mM peptide concentration without (full infill) and with (stripped infill) 240 μM of Dox. The *G*′ reported were taken at a frequency of 1 Hz and shear strain of 0.1% (frequency sweep curves for all samples are presented in Figure S1); (E) cumulative fraction of Dox released versus time (*t*).

With the exception of FK, all the other self-assembling peptides used have been the subject of previous studies by our group and have been shown to readily form β-sheet-rich fibers that entangle and associate into dense 3D fibrillar networks to form hydrogels (FE, ref (17); F8, ref (18); F8K, ref (19); KF8K, ref (8a)). In Figure 2A, the FTIR spectra obtained for the FK hydrogel is shown. As can clearly be seen, FK too is a strong β-sheet former, as evidenced by the presence of a strong band at 1624 cm^–1^ and a weaker band at 1694 cm^–1^, typical of the adoption by this family of peptides of β-sheet conformations. TEM (Figure 2B) confirmed the presence in FK too of thin fibers with radii ranging from 3 to 5 nm and the formation of a dense fibrillar network with junctions formed by the lateral association of these thin fibers into larger bundles.

Owing to the design used, this family of peptides forms antiparallel β-sheets with a hydrophobic face—where all the phenylalanine side groups are located—and a hydrophilic face—where all the lysine and glutamic acid residues side groups are located.^8a,8f,18^ The fibers formed are thought to result from the lengthwise association of two of these sheets to bury their hydrophobic faces resulting in the formation of β-sheet-rich fibers with rectangular cross-sections, as schematically shown in Figure 2C. There are two main fiber structural features any drug loaded in these hydrogels can interact with: the hydrophilic surfaces, rich in charged lysine and glutamic acid side groups (at pH 7), and the edges. The exact physicochemical properties of these edges depend on the peptide design and, in particular, on the position and number of lysine present at the peptide sequence ends.^8a^

In Figure 2D, the storage shear moduli, *G*′, obtained for all the hydrogels, formulated with and without 240 μM of Dox, are presented. This set of peptides leads to hydrogels with a broad range of mechanical properties from ∼30 Pa for FK to ∼40 kPa for FE. As discussed in detail in our previous studies, the bulk mechanical properties of these materials are affected by the design of the peptide used and the resulting fiber surfaces and physicochemical properties of edges. The detailed relationship between the peptide sequence and hydrogel mechanical properties has been the subject of a number of articles by our group^8a−8c,18,19^ and is outside the scope of this specific study. The incorporation of Dox clearly does not have a significant effect on the final *G*′ of the hydrogels, probably due to the low Dox-to-peptide molar ratio used, 0.017. Indeed, even if Dox interacts with the peptide fibers, as will be discussed below, its overall effect on the bulk mechanical properties of the hydrogels is expected to remain limited at the used molar ratio.

The release of Dox from the hydrogels was monitored by UV absorbance at 37 °C. 1 mL of PBS release media was added on the top of 500 μL of the hydrogel, and the Dox concentration in the media was measured at selected time points up to 72 h. No significant erosion or swelling of the hydrogels was observed over the time span investigated. In Figure 2E, the cumulative fraction of Dox released for each hydrogel is presented as a function of time, *t*. Two clear diffusion regimes can be seen: (I) *t* ≲ 17 h, with burst release followed by relatively fast Dox release, and (II) *t* ≳ 17 h, with slow or no Dox release. FE and KF8K show a significant Dox retention after 72 h, while F8 shows a significant release over the same time period. FK and F8K show similar intermediate behaviors, with Dox being released continuously, albeit at different rates across the time span investigated.

**Figure 3:**
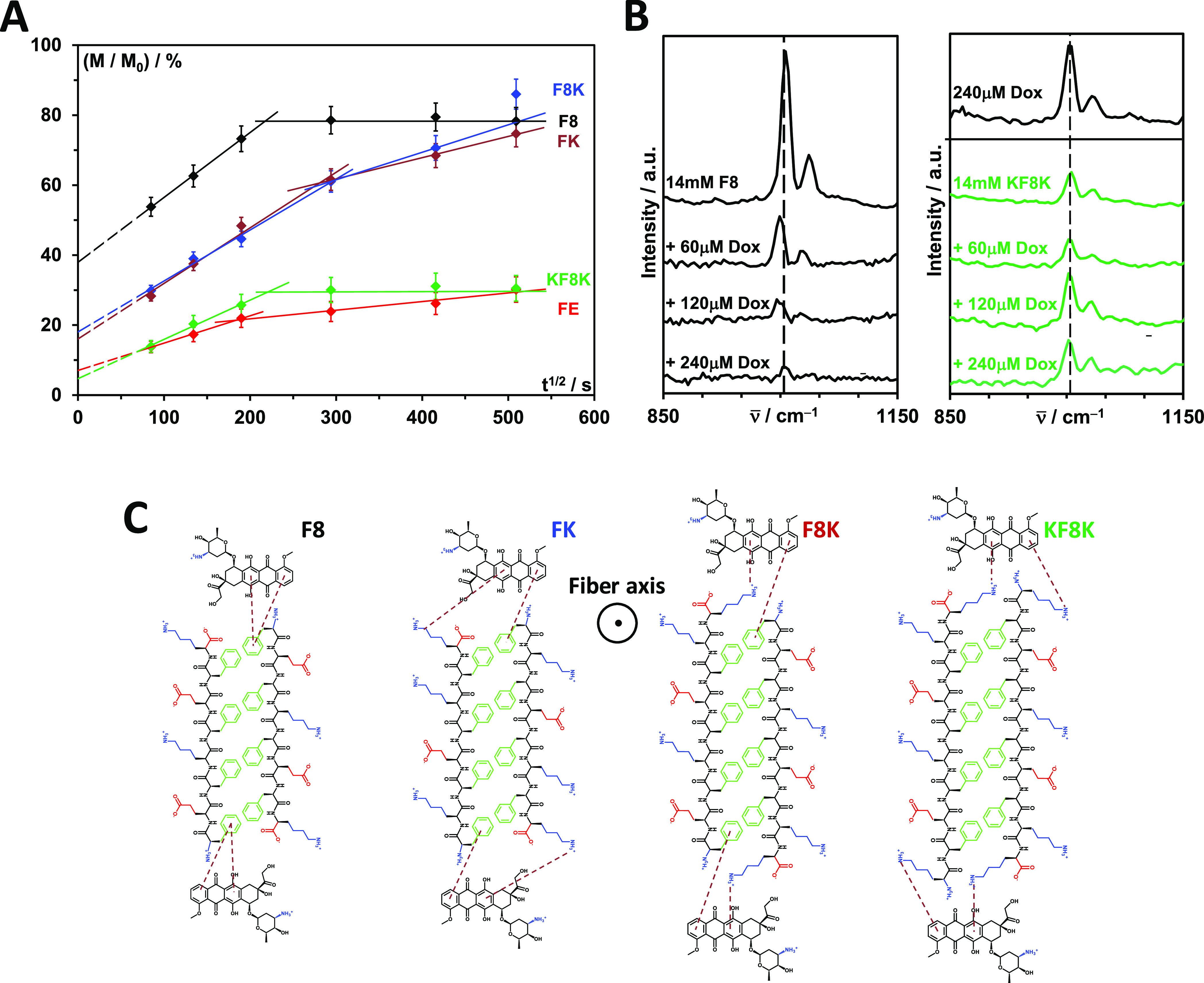
(A) Cumulative fraction of Dox releases versus *t*^1/2^ and best fits obtained using eq 2. All fitting parameters are listed in Table 1; (B) Raman spectra of Dox and F8 and KF8K hydrogels loaded with an increasing amount of Dox; (C) schematic representation of the potential nature of Dox interactions with the peptide fibers.

To extract quantitative data from the release curves, the cumulative fraction of Dox released was plotted as a function of *t*^1/2^ (Figure 3A). For both diffusion regimes, linear behaviors were observed. We, therefore, decided to use the non-Fickian diffusion model first proposed by Higuchi to analyze our data
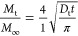
2where *M*_∞_ and *M*_*t*_ are the moles of Dox loaded into the hydrogel and released at time *t*, respectively, l is the thickness of the sample, in our case 8 mm, and *D*_*t*_ is the diffusion coefficient in m^2^ s^–1^. Although the model was originally developed by Higuchi to describe the dissolution and diffusion of a drug out of a matrix,^20^ it was subsequently shown by Rigter and Peppas to also apply to the diffusion of soluble drugs out of hydrogels slabs.^21^ One of the key assumptions in this model is that the drug is significantly smaller than the mesh size of the matrix. This is indeed the case as the mesh size of this family of peptide hydrogels was shown to range from 20 to 40 nm,^8a,18^ depending on the concentration, which is significantly larger than Dox molecular size, ∼1.2 nm. The best fits obtained using eq 2 are shown in Figure 3A. The amount of Dox released through burst release was estimated by extrapolating the linear fit of regime I (*t* < 17 h) to *t* = 0, while the time of diffusion regime change was taken as the crossover time between the linear fits of the two diffusion regimes. All the data extracted from the fitting of the release curves are summarized in Tables 1 and 2.

**Table 1:** Parameters Obtained from Fitting Dox Cumulative Release Curves Presented in Figure 3A Using eq 2

			diffusion regime I (*t* < 17 h)			diffusion regime II (*t* > 17 h)			
peptide	no. of K	charge	burst Dox released (%)	*D*_*t*_ (10^–7^ m^2^ s^–1^)	*R*2	*D*_*t*_ (10^–7^ m^2^ s^–1^)	maximum Dox released (%)	*R*2	time of diffusion regime change (h)
FE	1	–2	7 ± 1	0.77 ± 0.05	0.99	0.08 ± 0.01	30 ± 5^a^	0.96	9.4 ± 0.2
F8	2	0	38 ± 8	4.32 ± 0.18	0.99	~∼0	79 ± 8^b^		24.5 ± 0.3
F8K	3	+1	18 ± 5	2.66 ± 0.10	0.99	0.77 ± 0.06	86 ± 10^a^	0.96	18.6 ± 0.3
FK	3	+2	16 ± 5	3.17 ± 0.18	0.99	0.46 ± 0.05	75 ± 8^a^	0.99	21.9 ± 0.3
KF8K	4	+2	3 ± 1	1.97 ± 0.08	0.97	∼0	31 ± 5^b^		13.7 ± 0.3

a Maximum Dox released taken as Dox released after 72 h.

b Maximum Dox released taken as an average of Dox released at 24, 48, and 72 h.

**Table 2:** Fitting and Extracted Parameters Obtained from Fitting the Dox Cumulative Release Curves Presented in Figure 4 Using eq 2^a^

		diffusion regime I (*t* < 17 h)			diffusion regime II (*t* > 17 h)			
peptides	weight fraction of each peptide	burst Dox released (%)	*D*_*t*_ (10^–7^ m^2^ s^–1^)	*R*2	*D*_*t*_ (10^–7^ m^2^ s^–1^)	maximum Dox released (%)	*R*2	time of diffusion regime change (h)
F8/FE	75/25	19 ± 5	4.51 ± 0.14	0.98	0.09 ± 0.01	63 ± 9^b^	0.99	9.4 ± 0.2
F8/FE	25/75	0 ± 4	5.35 ± 0.14	0.99	0.23 ± 0.05	52 ± 7^b^	0.97	9.3 ± 0.2
F8/KF8K	99/1	28 ± 6	2.84 ± 0.10	0.99	∼0	56 ± 7^c^		9.7 ± 0.2
F8/KF8K	95/5	13 ± 5	1.92 ± 0.10	0.99	∼0	37 ± 5^c^		10.2 ± 0.2

a For pure systems F8, FE, and KF8K please see Table 1.

b Maximum Dox released taken as Dox released after 72 h.

c Maximum Dox released taken as an average of Dox released at 24, 48, and 72 h.

As mentioned above, FE and KF8K hydrogels are able to retain a significant fraction of Dox; for both hydrogels, after 72 h, only ∼30% of the drug has been released. In addition, the amount of Dox released through burst release is relatively low, ∼3 and ∼7%, respectively. Clearly, for both systems, strong interactions are present between peptide fibers and drug molecules. For FE, as discussed in our previous study, it is thought that at pH 7, strong electrostatic interactions between the negatively charged fibers and the positively charged drug molecules exist, leading to Dox retention.^5e,17a^ For KF8K, the fibers carry a positive charge and, therefore, electrostatic interactions are repulsive in nature and cannot lead to drug retention. Instead, in this case, strong cation−π interactions are thought to be present between the lysine end-residues and Dox aromatic rings. From the F8 Dox release curve, it is clear that when these two lysine end-residues are absent, although F8 is neutrally charged, interactions between Dox and peptide fibers are weak, resulting in a significant burst release, ∼40% and fast diffusion with 80% of Dox being released within ∼13.5 h. It should be noted that due to the experimental setup used (volume fractions: 1/3 hydrogel slab at the bottom and 2/3 PBS release media on the top), if a simple 2/3 dilution is assumed, the maximum fraction of Dox released that would be expected is 67%. It is, therefore, reasonable to assume that after ∼13.5 h, the release buffer on the top of the hydrogel is saturated, resulting in diffusion stopping. Dox is clearly not able to interact with the lysine present on the surface of F8 fibers, which are adjacent to a glutamic acid. We hypothesize that these carboxylic acid side groups interfere electrostatically and prevent the establishment of strong cation−π interactions between the lysine amine side groups and Dox aromatic rings.

These results point toward the key role played by the fiber edges in the release of Dox. As discussed in our previous study, F8 and KF8K have fiber edges with very different physicochemical properties. In F8 fibers, the first phenylalanine side group has been shown to be exposed to the media creating a hydrophobic fiber edge rich in exposed aromatic rings, while in KF8K, the presence of the two terminal lysine results in the first phenylalanine side group being buried within the β-sheet fiber leading to a hydrophilic fiber edge rich in amine groups.^8a^

The difference in the nature of interactions between F8 and KF8K fibers and Dox was confirmed by Raman spectroscopy. In Figure 3B, the Raman spectra of Dox, F8, and KF8K are shown. In all cases, a strong band at 1002 cm^–1^ and a weaker band at 1031 cm^–1^ were observed, which have been assigned in the literature to the breathing vibration mode of aromatic rings and the C–H groups present on them, respectively.^22^ For KF8K, the bands were found to be significantly weaker compared to F8, pointing toward a very different environment surrounding the phenylalanine aromatic rings. Indeed, as discussed above, for KF8K, the presence of the two terminal lysine results in the first phenylalanine side group being buried within the β-sheet fiber, leading probably to steric confinement and dampening of the aromatic vibrations.

When Dox is added to F8 hydrogels, the intensities of the aromatic bands decrease significantly, and a downshift in their positions was observed, pointing toward interactions being present between Dox and the F8 fiber edges.^23^ These interactions can be potentially hydrophobic or π–π in nature. In either case, they are weak and unstable as they do not lead to any significant retention of the drug. This is confirmed when the amount of drug loaded is decreased, as even for low loading levels, no significant retention of the drug was observed (Figure S2A). For KF8K, the addition of Dox neither significantly affects the intensities nor the positions of the aromatic bands, confirming the absence in this case, as expected, of direct interactions between Dox and the first phenylalanine aromatic side group. On the other hand, when added, Dox did not contribute to the aromatic band’s overall intensities observed for the KF8K hydrogel, suggesting the presence of cation−π interactions. Indeed, as shown in Figure S3, when Boc-K-Me is added to a 240 μm Dox solution, the intensities of the 1002 and 1031 cm^–1^ bands decrease, confirming the presence of strong cation−π interactions between the lysine amino side group and Dox aromatic rings.

For F8K and FK, Dox was released continuously over 72 h. The decrease in the amount released through burst release and the decrease in initial diffusion rates compared to F8 suggest an increased level of interactions between peptide fibers and Dox in these two systems even though both peptides at pH 7 carry positive charges (see Table 1). These results suggest that Dox is able to interact with the end-lysine (Figure 3C), which in both these peptides is not in direct proximity of a glutamic acid side group. For FK, the end-lysine is placed on the same side of the β-sheet plane as the other hydrophilic side groups (E and K), while in F8K, it is placed on the same side of the β-sheet plane as the phenylalanine (F) hydrophobic side groups (Figure 3C). As both systems behave similarly, we hypothesize that this end group’s position is very “flexible,” allowing them to interact with Dox in a similar fashion. FK and F8K also have phenylalanine as their starting amino acid and, therefore, fiber edges rich in exposed phenyl rings that clearly prevent the formation of stable cation−π interactions, resulting in complete release of Dox in this case within 72 h. The decrease in diffusion rates after ∼24 h is probably due to the saturation of the release media on the top of the hydrogel. As seen for F8, even when the amount of Dox loaded was decreased, no significant drug retention was observed (Figure S2), confirming, for these two systems too, the absence of stable interactions forming between peptide fibers and Dox.

In Figure 3C, a schematic representation of the fiber cross-sections and fiber–drug potential interactions are shown. Clearly, whether due to the fact that it results in the first phenylalanine side group not being exposed or in a molecular arrangement of the end-lysine that promotes strong cation−π binding of Dox, the presence of two lysine residues, one at each end of KF8K, is key to the retention of Dox within this peptide hydrogel.

**Figure 4:**
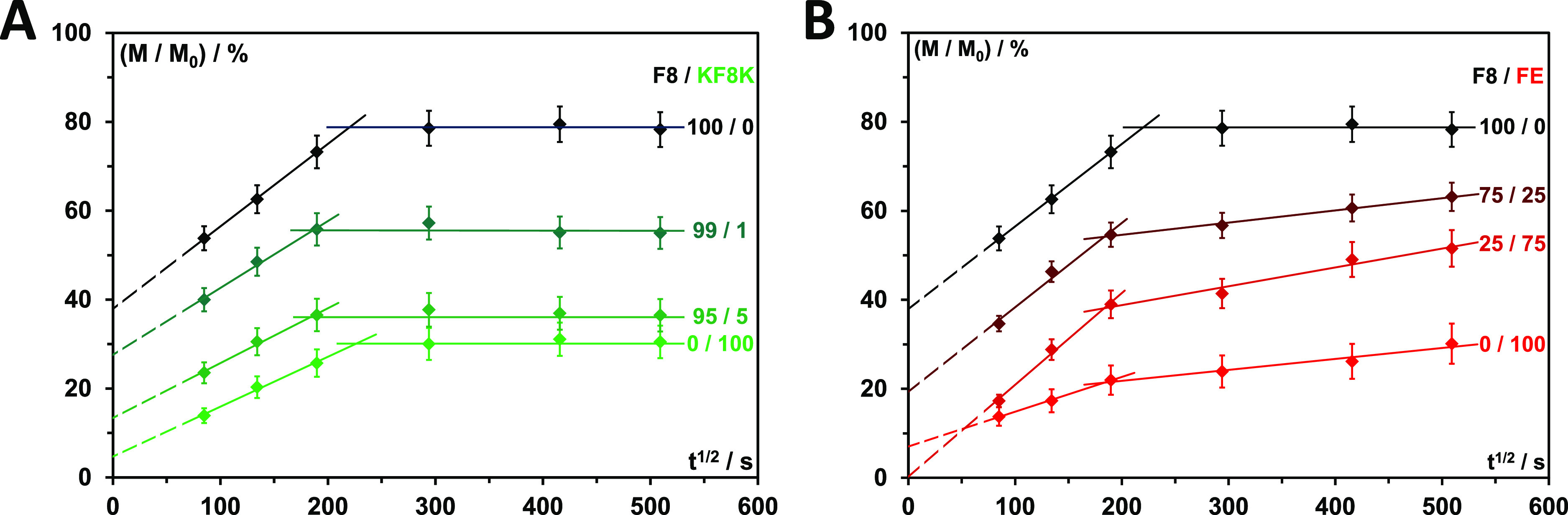
Cumulative fraction of Dox releases versus *t*^1/2^ and best fits obtained using eq 2 for F8/KF8K (A) and F8/FE B) blends. All fitting parameters are listed in Table 2.

For both FE and KF8K hydrogels, when Dox loading is decreased to 120 μM, no drug release was observed over the timespan investigated (data not shown). This observation, combined with the fact that both systems show fast release of 20 and 30% of Dox, respectively, during the first 17–20 h, suggests the presence of slightly different interaction stoichiometries in these two systems: for FE at 14 mM concentration ∼200 μM Dox is bound, while for KF8K ∼170 μM Dox is bound. Cation−π and electrostatic interactions are very different in nature. Electrostatic interactions are long-range and diffusive, while cation−π interactions are short-range and molecular. The different nature of these interactions is highlighted by the fact that for FE, slow diffusion of Dox is still observed at later times (Regime II), while for KF8K, Dox seems permanently (on the timescale investigated) bound and retained. This difference in type of interactions is even more pronounced when FE and KF8K are added to F8 to create composite hydrogels (Figure 4). For FE, the amount of Dox retention is roughly proportional to the amount of FE added and, therefore, to the amount of negative charge present on the fibers, while for KF8K, the addition of 5% of the peptide to F8 leads roughly to the same level of Dox retention, as observed for pure KF8K hydrogels clearly showing the molecular nature of cation−π interactions (Figures 4 and S4). 5% addition of KF8K corresponds to the addition of 700 μM end-lysine and, therefore, to an end-lysine/Dox molecular ratio of ∼3 (Figure 4A). Interestingly, the stoichiometry discussed above (170 μM Dox bound in KF8K 14 mM hydrogel) clearly relates to the amount of “surface area” available for Dox biding to the fibers as the addition of further end-lysine does significantly change the maximum amount of Dox bound at this concentration.

**Figure 5:**
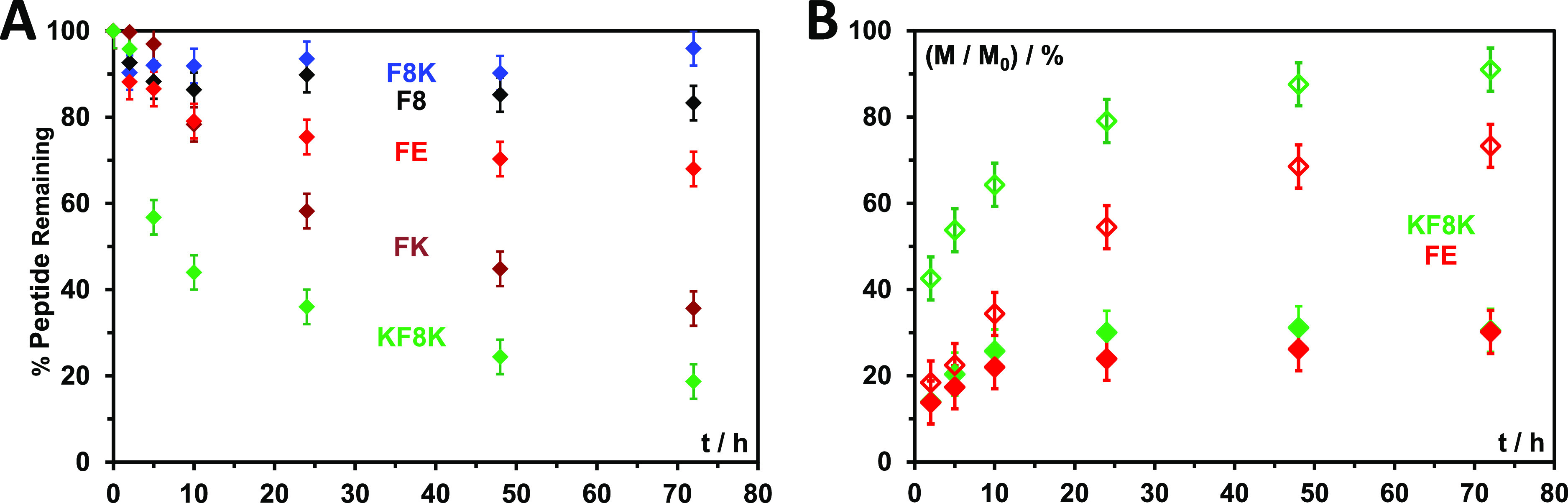
(A) Fraction of nondegraded peptides versus time (*t*); (B) Cumulative fraction of Dox releases versus time obtained for FE and KF8K using PBS (solid symbols) and 50/50 FBS/PBS media mixture (open symbols) as supernatant. (F8, FK, and F8K release curves are shown separately in Figure S5 for the ease of visualization).

We also examined the susceptibility of these peptide hydrogels to enzymatic degradation by incubating them in a 50 FBS/50 PBS media mixture. At the selected time points, the samples were homogenized (release buffer + remaining hydrogel), and the fraction of intact, that is, nondegraded peptide was estimated via HPLC. FBS contains a wide array of proteases and other enzymes that will degrade proteins and peptides. These hydrogels show a broad range of susceptibilities to enzymatic degradation (Figure 5A), with F8K being the least susceptible while KF8K is the most susceptible. Dox release was also measured using a 50 FBS/50 PBS mixture as release media. For F8 and F8K hydrogels, as expected, very similar release profiles were obtained in the presence and absence of FBS (Figure S5), as both these hydrogels show a minimal degradation. FK hydrogels, on the other hand, showed a slight increase in Dox release at the early time point, probably due to the low level of degradation observed up to 12 h. From 24 h onward, similar release profiles were obtained in the presence and absence of FBS, probably due to the fact that by then most of the Dox has been released, and the supernatant release media is reaching saturation (Figure S5).

As discussed above, FE and KF8K hydrogels can retain a significant amount of drug and, therefore, for these two systems, degradation has a significant impact on the release curves obtained. For FE, low levels of degradation are observed during the first 12 h resulting in a small increase is Dox released during the initial stage (Figure 5B). Hydrogel degradation becomes more substantial at later times (>24 h), resulting in a significant increase in the Dox released. For KF8K, a substantial increase in the Dox released is observed even at early time points (Figure 5B) due to the fast degradation of this hydrogel, ∼40% of the peptide being already degraded after 5 h (Figure 5A).

**Figure 6:**
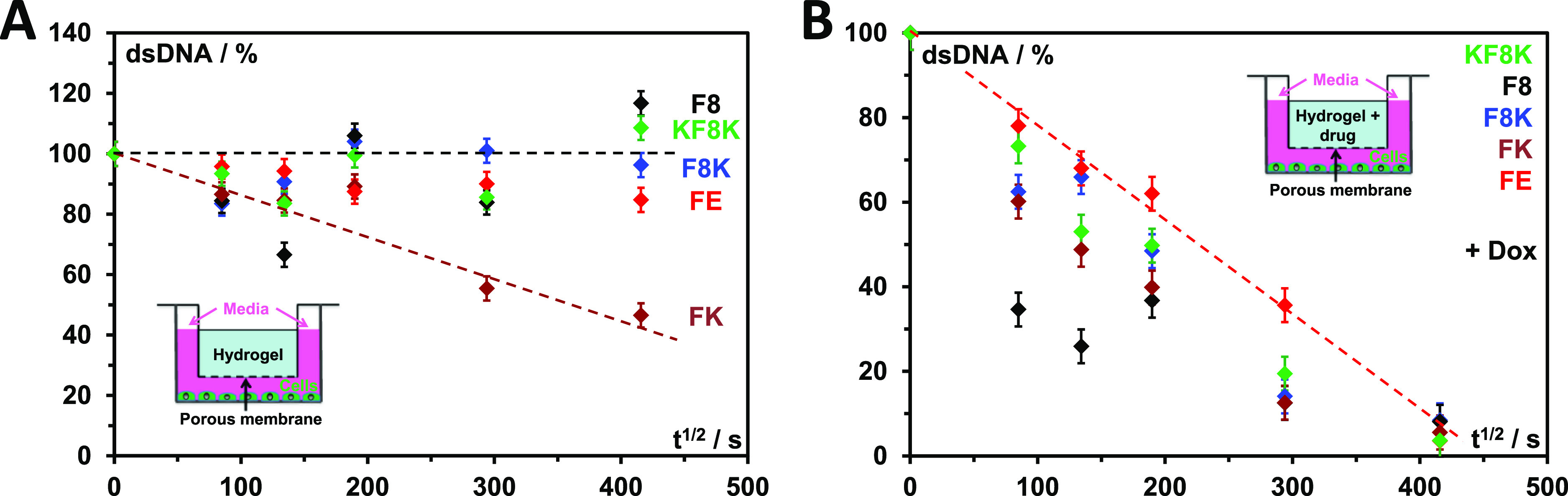
Fraction of dsDNA versus *t*^1/2^ obtained for 3T3 murine fibroblast cultured in presence of: (A) hydrogels (14 mM) and (B) Dox loaded (240 μm) hydrogels.

Finally, we confirmed that the Dox released from the hydrogels was still active by investigating its effect on 3T3 mouse fibroblasts. This was achieved by culturing the fibroblasts in 2D in cell culture wells and placing the hydrogels (loaded and non-loaded with Dox) in inserts equipped with porous membranes above the cells (see schematics in Figure 6). This experimental setup was used to avoid a direct contact between the hydrogels and the cells but still allow the drug to diffuse from the hydrogels into the cell culture media. Dox is known to affect cell proliferation and viability via DNA intercalation and inhibition of topoisomerase II.^24,25^ Therefore, at the selected time points, the effect of Dox on fibroblasts viability was evaluated by measuring the total amount of double-stranded DNA (dsDNA), which is directly proportional to the number of live cells present.

First, non-loaded hydrogels were used as control. As can be seen from Figure 6A, good cell viabilities were observed for FE, F8, F8K, and KF8K hydrogels over 48 h, as the amounts of dsDNA were found to remain roughly constant. It should be noted that a minimal proliferation is expected to be observed over 48 h for these types of cells under the used conditions (no media changes). On the other hand, FK clearly showed some level of cytotoxicity, as the amount of dsDNA detected after 48 h was only 40% of the amount measured at time *t* = 0. As the media used contained 10% FBS, to ensure cell viability over 48 h without media changes, the expectation is that some small amounts of the peptide and/or its degradation products did diffuse in the cell culture media over time. Clearly, FK and/or its degradation products present some level of cytotoxicity. The exact relationship between the peptide sequence and cytotoxicity is beyond the scope of the current study.

Next, the hydrogels were loaded with 240 μM Dox. As can clearly be seen from Figure 6B, the amount of dsDNA measured for all five systems decreases significantly over time, and after 48 h, no significant number of live cells were found. Interestingly, the amount of dsDNA measured after 2 h is a direct reflection of the amount of Dox released during the burst-release phase (Figure 3A and Table 1), with F8 showing the lowest and FE the highest cell viability.

## Conclusions

We have investigated how the design of a short β-sheet-forming peptides can be modified to control the release of the oncologic drug Dox. Different types of interactions can be used to control the retention and release of small drug molecules from hydrogels. In this study, electrostatic and cation−π interactions were used, as Dox is positively charged at pH 7 and contains aromatic moieties. In the case of electrostatic interactions, the use of a peptide carrying a negative charge allowed the retention and slow release of Dox. The amount of drug retained in F8/FE composite hydrogels was found to be directly proportional to the amount of charge (ratio of the FE peptide present) carried by the peptide fibers. The position and number of the end-lysine were found to play a key role in the retention of Dox when cation−π interactions were used. In this case, the amount of Dox retained in F8/KF8K composite hydrogels was linked to the amount of end-lysine introduced, and an end-lysine/Dox interaction stoichiometry of 3/1 was obtained. In both cases, FE and KF8K, the maximum amount of Dox retained was also found to be related to the overall concentration of the hydrogels and, therefore, to the overall fiber surface area available for interaction with the drug. For 14 mM hydrogel, ∼170–200 μM Dox could be retained after 24 h. For FE, slow release at longer time points was observed, while for KF8K, the drug was found to be bound, and no further release was observed over the timespan investigated. The susceptibility of the hydrogel to enzymatic degradation was also investigated. This set of peptides shows a broad range of susceptibilities opening the prospect of being able to control also the rate of enzymatic degradation of these hydrogels. Finally, the Dox released from the hydrogel was shown to be active and affect 3T3 mouse fibroblasts viability in vitro. Our study clearly shows the potential of this peptide design as a platform for the formulation of injectable and sprayable hydrogels for controlled drug delivery.

## Supporting Information Available

Frequency sweeps of Dox-loaded and nonloaded hydrogels; cumulative fraction of Dox released for F8, F8K, and FK; Raman spectra of Dox/Boc-lysine-methyl solutions; fraction of Dox released in mixed F8/FE and F8/KF8K composite hydrogels; cumulative fraction of Dox released using PBS and FBS/PBS (PDF)
