# Supplementary material for: Controlling Doxorubicin Release from a Peptide Hydrogel through Fine-Tuning of Drug–Peptide Fiber Interactions
Source: Biomacromolecules. 2022 May 11;23(6):2624–34. doi: 10.1021/acs.biomac.2c00356 (PMC9198986; doi:10.1021/acs.biomac.2c00356)
Supplement: Supplementary file 1 — bm2c00356_si_001.pdf [file bm2c00356_si_001.pdf]

Supplementary Information

Controlling doxorubicin release from peptide hydrogel through fine-tuning drug-peptide fibre interactions

Mohamed A. Elsawy [1,2,§], Jacek K. Wychowaniec [1,2,ξ], Luis A. Castillo Díaz [1,2,δ], Andrew M. Smith [1,2], Aline F. Miller [3,2], Alberto Saiani [1,2,*]

[1] Department of Materials, University of Manchester, Oxford Road, Manchester M13 9PL, UK

[2] Manchester Institute of Biotechnology, Oxford Road, Manchester M13 9PL, UK

[3] Department of Chemical Engineering and Analytical Sciences, University of Manchester, Oxford Road, Manchester M13 9PL, UK

Current addresses:

[§] Leicester Institute for Pharmaceutical Innovation, Leicester School of Pharmacy, De Monfort University, The Gateway, Leicester LE1 9BH, UK

[ξ] AO Research Institute Davos, Clavadelerstrasse 8, 7270, Davos, Switzerland

[δ] Departamento de Medicina y Ciencias de la Salud, División de Ciencias Biológicas y de la Salud, Universidad de Sonora, Hermosillo, México

[*] Corresponding author: Phone: +44 161 306 5981; e-mail: a.saiani@manchester.ac.uk

Supplementary Figures

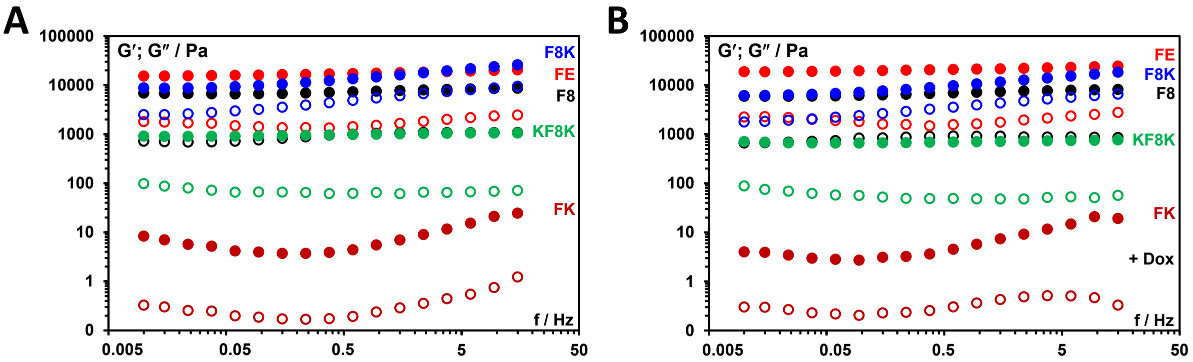

Figure S1: Storage (G'- closed symbols) and loss (G"- open symbols) shear moduli vs frequency (f) curves obtained at 0.1 % strain for hydrogels formulated at 14 mM peptide concentration: ***A)*** *without and* ***B)*** *with 240 μM of Dox.*

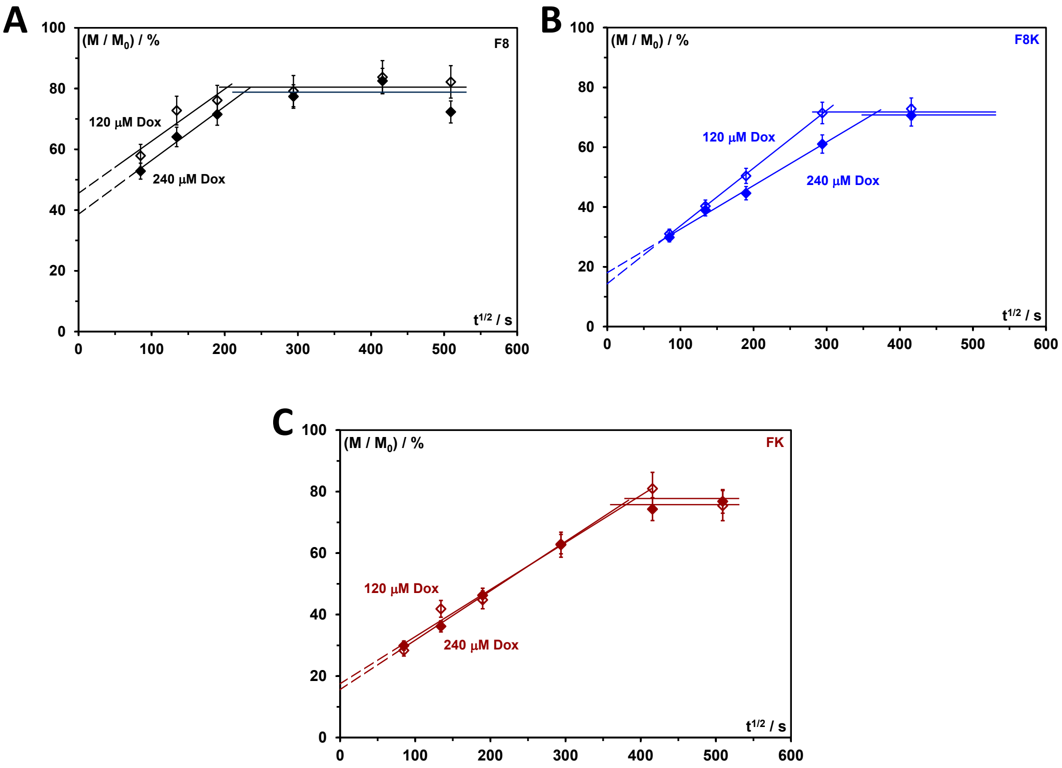

Figure S2: Cumulative fraction of Dox releases vs $t^{1/2}$ for F8 (A), F8K (B) and FK (C) loaded with 120 μM (open symbols) and 240 μM (close symbols) of Dox.

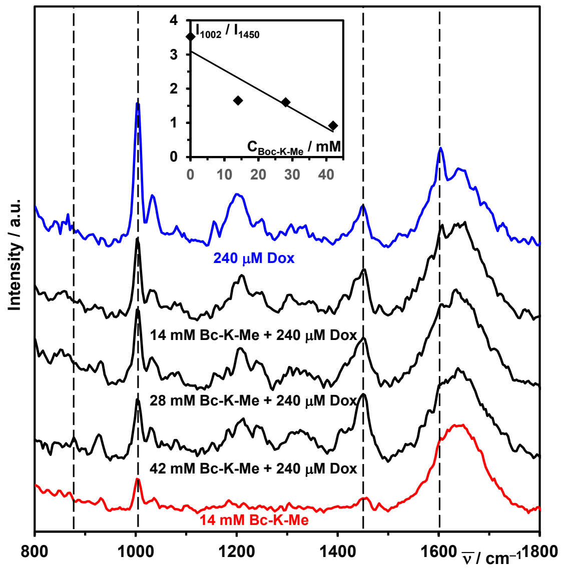

Figure S3: Raman spectra of 240 μm Dox / Boc-K-Me mixtures and of 42 mM Boc-K-Me solution. Insert: 1002 over 1450 cm^{-1} Raman bands intensity ratio of 240 μm Dox / Boc-K-Me mixtures vs Boc-K-Me concentration.

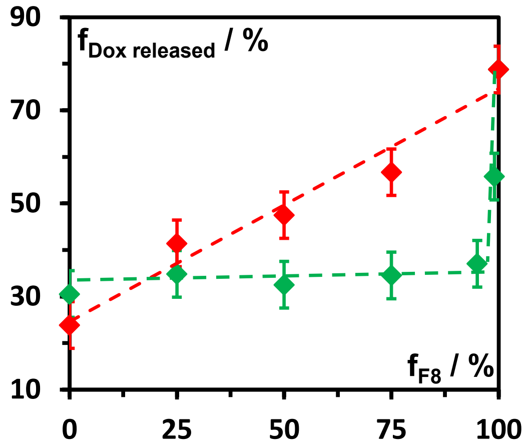

Figure S4: Fraction of Dox released ($f_{Dox\ released}$) vs fraction of F8 peptide used to formulate the hydrogels (f_{F8}) for F8/FE (red symbols) and F8/KF8K (green symbols) composite hydrogels loaded with 240 μM (close symbols) of Dox. For F8/FE hydrogel $f_{dox\ released}$ was taken as the fraction of Dox released at 24 h while for F8/KF8K composite hydrogels it was taken as the average of dox release at 24, 48 and 72 h.

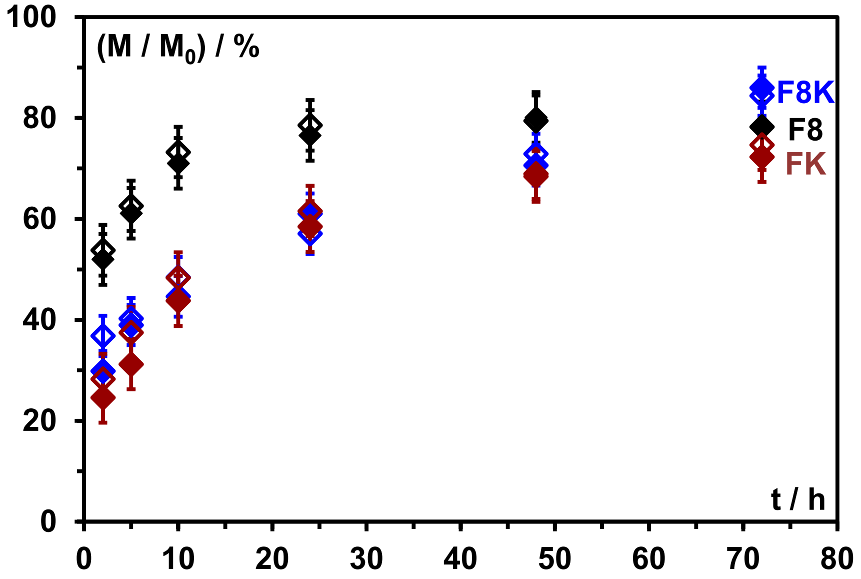

Figure S5: Cumulative fraction of Dox releases vs time obtained for F8, FE and KF8K using PBS (solid symbols) and 50 FBS / 50 PBS (open symbols) media mixture as supernatant.
